# Impacts of climate change on basin vegetation based on Biome-BGC model: A case study with the Jialing River Basin

**DOI:** 10.1371/journal.pone.0335685

**Published:** 2026-02-13

**Authors:** Chuansen Wu, Xiaoning Hou, Shanghong Zhang, Weiyi Zhou, Yang Zhou

**Affiliations:** School of Water Resources and Hydropower Engineering, North China Electric Power University, Beijing, China; Euro-Mediterranean Center for Climate Change: Fondazione Centro Euro-Mediterraneo sui Cambiamenti Climatici, ITALY

## Abstract

Vegetation, as a key component of land cover, plays a vital role in regulating energy exchange and water balance at different spatial and temporal scales. It is thus important to explore dynamic processes of changes in vegetation cover under changing environmental conditions in the context of global climate change. In this study, the Jialing River Basin (JRB) was selected as a case study, with the leaf area index (LAI) used as the primary indicator to represent JRB vegetation cover and growth status. The Biome-BGC model was employed to simulate the growth of various vegetation types within the basin. We calibrated the optimal range of multiple physiological and ecological parameters of vegetation and analyzed vegetation responses to climate change. The results showed that under four CMIP6 climate scenarios (SSP126, SSP245, SSP370, and SSP585), both temperature and precipitation in the basin are projected to increase. From 1976 to 2016, the vegetation coverage of the basin remained high, and on a monthly timescale, the grasslands are more responsive to climate-induced variability than woodlands. Under the influence of a warmer, more humid climate from 2023 to 2100, the LAI of vegetation in the basin is projected to show an increasing trend, and the vegetation coverage of woodland will still exceed that of grassland. These findings contribute to a more accurate simulation of vegetation dynamics under climate change and can inform the development of effective vegetation conservation and management strategies.

## 1. Introduction

Vegetation (referring generally to terrestrial plant communities) is a key component of land cover and is closely linked to global energy and biochemical cycles [1]. Vegetation growth status is related to the health of terrestrial ecosystems and plays vital roles in regulating global carbon balance, mitigating the atmospheric greenhouse effect, and maintaining biodiversity. Moreover, vegetation has important effects on seasonal, annual, and decadal energy exchange; water balance; and the terrestrial carbon cycle at different spatial scales from regional to global [2–7]. Vegetation is in large part responsible for connecting soil, atmosphere, hydrology, and other ecological elements, and thus it provides a strong basis for the stability of natural ecosystems, biodiversity, and human activities [8–11]. In parallel, changes in vegetation dynamics are often used as key biological indicators of Earth’s climate change because of the extreme sensitivity of vegetation to such change [12–16]. Therefore, in the context of increasing changes in global climate, it is necessary to understand the characteristics of vegetation dynamics under varying climatic conditions in order to uncover general ecosystem patterns and to inform regional environmental assessment and protection strategies.

With continuing developments in satellite remote sensing technologies, most studies on vegetation dynamics are now based on remote sensing data, using a variety of statistical analysis methods and relatively simple light utilization efficiency models such as the CASA model to investigate the spatial and temporal evolution trends of vegetation in different periods [17]. For example, Ranjan et al. applied the Mann–Kendall trend analysis method to assess the vegetation greening rates in eastern India and demonstrated a significant decline in vegetation cover from 2000 to 2004 due to mining activities [18]; Liu et al. simulated the response of vegetation to meteorological factors in the study area with a CASA model constructed using data from remote sensing images [19]; and Zhang et al. found using the Hurst index that vegetation degradation may occur in the future in some areas of the Pearl River Delta region if the existing ecological planning is maintained [20].

Although there have been many types of research on vegetation cover change, including the use of statistical methods and remote sensing-based analyses, many of these approaches primarily rely on long time historical vegetation data and do not incorporate the underlying ecological processes [21,22]. Specifically, statistics-based models often fail to capture the physiological mechanisms of vegetation growth, which may lead to considerable forecasting uncertainties, particularly under rapidly changing external factors such as climate and soil conditions. This limitation underscores the importance of using process-based models.

Compared to statistical analysis methods, process-based models such as HYBRID, MAPSS, and Biome-BGC place greater emphasis on the physical mechanisms of ecosystem evolution, and have been widely applied in vegetation dynamics research [23–26]. As a physiological and ecological model with daily time steps, Biome-BGC comprehensively accounts for the physiological and ecological characteristics of different vegetation types at a regional scale, as well as the cycles for carbon, nitrogen, and water; soil processes and ecosystem process energy flows; and integrates the effects of external factors such as climate, soil, and human activities. Furthermore, Biome-BGC demonstrates strong capability in simulating the vegetation dynamics under changing environmental conditions. This model has been successfully applied to the estimation of regional vegetation productivity [27–30], simulation of ecosystem processes under different climatic conditions [31–33], and simulation of the impacts of disturbance and management on ecosystems [34–36]. Although Biome-BGC is a mechanistic model that can simulate the processes of plant growth and vegetation evolution well, a large number of physiological and ecological parameters is required to simulate ecosystem development processes based on clear mechanistic principles, and the process of parameter calibration is lengthy [37–39]. In addition, most current applications of this model primarily focus on the carbon and water cycles in small regions at interannual scales. However, studies on the influence of vegetation evolution trends on hydrological processes of large basins at monthly scales remain limited. Therefore, optimizing the physiological and ecological parameters for vegetation in large basins—so as to simulate and predict the growth under varying environmental conditions—remains a challenge. Addressing this issue is essential for supporting future research on hydrological changes at the basin scale.

In this study, we used the leaf area index (LAI) to characterize vegetation cover and growth status in the Jialing River Basin (JRB). Using the Biome-BGC model, we simulated the growth of different vegetation types, calibrated reasonable ranges for physiological and ecological parameters of vegetation in the basin, and explored how vegetation cover in the basin may respond to climate change. The findings of this study can provide a scientific basis for decision makers to develop effective ecological and environmental protection strategies.

## 2. Materials and methods

### 2.1. Study area

**Fig 1:**
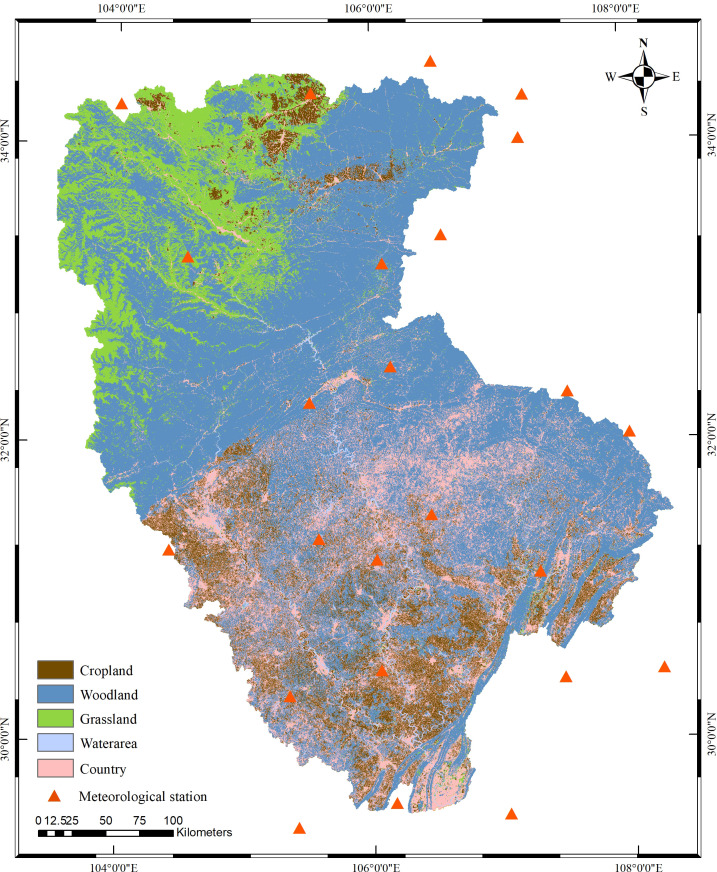
Jialing River Basin map.

The Jialing River, originating from Qinling Mountains in Shaanxi Province, China, is a major tributary of the upper reaches of the Yangtze River. Influenced by the East Asian monsoon, the summer climate in the JRB is warm and humid, with precipitation concentrated during this season. The average annual rainfall is 872 mm, and the average annual daily maximum and minimum temperatures are 20.1 °C and 11.3 °C, respectively. The watershed exhibits a significant elevation gradient, with the highest point reaching 5,556 meters and the lowest only 67 meters. The terrain flattens progressively from upstream to downstream, forming a spatial distribution pattern in which forests (collectively referred to as woodlands) and grasslands dominate the upstream and midstream areas, while farmland predominates downstream. Due to the favorable climate, a large area, and complex topography, more than 90% of the basin is covered by vegetation [40]. In addition to crops, the region supports various vegetation types including deciduous broadleaf forest, evergreen broadleaf forest, and coniferous–broadleaf mixed forest (Fig 1).

The maps were generated by ArcGIS 10.8.2 and were for illustrative purposes only. The licensing and data use information can be found at: https://livingatlas.arcgis.com/landcoverexplorer/, and the data are provided under the CC BY 4.0 license.

### 2.2. Data used

The meteorological data used in this study consist of observed records from 24 meteorological stations within the JRB, CMIP6-simulated meteorological data, and atmospheric CO_2_ concentration data. The vegetation data include LAI (leaf area index) for the JRB. The sources and accuracy of all datasets are summarized in Table 1.

**Table 1:** Sources of data for the study and the data accuracy.

Data type	Name	Time	Accuracy	Source
Meteorological data	Historical precipitation, maximum temperature, minimum temperature	1975–2016	Daily	http://data.cma.cn/
	Simulated precipitation and temperature by CMIP6	1975–2100	Daily	https://esgf-node.llnl.gov/search/cmip6/
	Carbon dioxide mole fraction of dry air	1975–2100	Daily	https://gml.noaa.gov/
Vegetation cover data	Leaf area index	1998–2016	0.05 degree	http://www.glass.umd.edu/

### 2.3. Vegetation growth model

As a physiological and ecological process-based model, Biome-BGC comprehensively accounts for the physiological traits of different vegetation types, the carbon, nitrogen, and water cycles; soil processes and energy flows associated with ecosystem functioning. It is capable of simulating carbon, nitrogen, and water fluxes in terrestrial ecosystems at various spatial scales with a daily time step [41–43]. The input files for Biome-BGC include a control file, a meteorological driver file, and a vegetation physiological and ecological parameters file. The control file mainly contains information such as latitude, longitude, elevation, vegetation type, interannual variation of CO_2_ concentration, soil mechanical composition, and effective depth of the study area. The meteorological driving data mainly include daily maximum temperature, minimum temperature, precipitation, vapor pressure deficit, solar radiation, and day length. There are more than 40 physiological and ecological parameters, including vegetation phenology, leaf carbon-to-nitrogen (C:N) ratio, canopy extinction coefficient, specific leaf area, maximum stomatal conductance, and canopy water interception coefficient. The simulation process includes two modes: Spin-up and Normal. Spin-up mode is designed to iteratively simulate vegetation growth based on pre-set physiological and ecological parameters together with meteorological inputs until the model state variables converge to equilibrium, which is regarded as the stable state of the ecosystem. Normal mode typically simulates the vegetation growth for a target year based on the equilibrium conditions established during the Spin-up phase. The main algorithms used in Biome-BGC are as follows (Table 2) [41–43]:

**Table 2:** Parameter meanings in the formula (1)-(8).

Parameter	Meaning	Parameter	Meaning
$V_{j}$	maximum photosynthetic rate as influenced by temperature	$V_{max}$	maximum rate of photosynthesis when the precipitation and temperature reach the ideal conditions
$T_{min}$	daily minimum temperature	ω	soil-water pressure potential
$V_{n}$	mean photosynthetic rate per unit leaf area	LAI	leaf area index
β	canopy extinction coefficient	∂	quantum yield
PAR	photosynthetically active radiation	P	net photosynthesis after deducting autotrophic respiration and heterotrophic respiration
D	sunshine duration	δ	ratio of maximum photosynthesis time to total sunshine duration
$R_{s}$	total autotrophic respiratory of vegetation	$R_{x,j}$	maintenance respiratory consumption of vegetation stems, leaves, fine roots, and coarse roots
$R_{g}$	Carbon consumption for vegetation growth and respiration	$M_{i}$	biomass of vegetation components
$r_{i}$	maintenance respiratory coefficient	$T_{\text{1}\text{0}}$	temperature factor
$T_{a}$	air temperature	$T_{b}$	root zone temperature
$r_{g}$	growth and respiration coefficients of vegetation components	$R_{h}$	total amount of gas released by microorganisms during decomposition
$k_{i}$	maximum decomposition rate of microorganisms	$L_{C}$	effect of lignin content
A	soil temperature and moisture effects	$C_{i}$	carbon storage
$T_{m}$	soil effect on organic matter transformation	i=1,…,8	Represents surface structure, soil structure, active organic matter, microorganisms, surface metabolites, soil metabolites, slowly decomposing organic matter, and passively decomposing organic matter, respectively
L	organic matter dispersed by vegetation into the soil	Q	total amount of carbon stored in vegetation
$R_{l}$	proportions of leaves in total vegetation carbon	$R_{r}$	proportions of roots in total vegetation carbon
$R_{s}$	proportions of stems in total vegetation carbon	$t_{l}$	average survival time of leaves
$t_{r}$	average survival time of roots	$t_{s}$	average survival time of stems

(1) Photosynthesis model

$$V_{j}=V_{max}f\left(T_{min}\right)f(\omega )$$ (1)

$$V_{n}=\left(\frac{V_{j}}{\beta }LAI\right)ln\left(\frac{V_{j}+\partial \cdot PAR}{V_{j}+\partial \cdot PAR\cdot \sqrt{-\beta LAI}}\right)$$ (2)

$$P=V_{j}\cdot LAI\cdot \delta D$$ (3)

(2) Autotrophic respiration

$$R_{s}=\sum_{i=\text{1}}^{\text{4}}R_{x,j}+R_{g}$$ (4)

$$R_{x,j}=M_{i}r_{i}T_{\text{1}\text{0}}^{\left(T_{a}-T_{b}\right)/\text{1}\text{0}}$$ (5)

$$R_{g}=r_{g}(GPP)$$ (6)

(3) Heterotrophic respiration

$$R_{h}=\sum_{i=\text{1},\text{2}}k_{i}L_{C}AC_{i}+\sum_{i=\text{3}}k_{i}AT_{m}C_{i}+\sum_{i=\text{4}}^{\text{8}}k_{i}L_{C}AC_{i}$$ (7)

(4) Total vegetation litterfall

$$L=\frac{R_{l}Q}{t_{l}}+\frac{R_{r}Q}{t_{r}}+\frac{R_{s}Q}{t_{s}}$$ (8)

The MT-CLIM (Mountain Microclimate Simulation Model) is a daily-scale climate simulation model based on terrain climatology and diurnal climate theory [44]. Its basic approach incorporates factors such as elevation, slope, and aspect, which affect microclimatic variation. By using measured site-level meteorological data such as daily temperature and precipitation, MT-CLIM simulates and estimates daily values of radiation, humidity, and other meteorological variables for the target location. Because of the difficulty in obtaining measured data for solar radiation, vapor pressure deficit, and day length in the study area required by the Biome-BGC model, we used MT-CLIM to estimate the aforementioned data.

(1) Vapor Pressure Deficit

$$VPD=e_{s}-e_{m}=e_{\text{0}}exp\left(\frac{\text{1}\text{7}.\text{2}\text{6}\text{9}T_{ave}}{\text{2}\text{3}\text{7}.\text{3}+T_{ave}}\right)-e_{\text{0}}exp\left(\frac{\text{1}\text{7}.\text{2}\text{6}\text{9}T_{dew}}{\text{2}\text{3}\text{7}.\text{3}+T_{dew}}\right)$$ (9)

where *e*_*s*_ is the saturation vapor pressure; *e*_*m*_ is the actual vapor pressure; *e*_*0*_ is the saturation vapor pressure at 0 °C (6.1078 kPa); *T*_*ave*_ is the daily average air temperature; *T*_*dew*_ is the corrected dew point temperature.

(2) Radiation

$$\text{Diffuse radiation}\to R_{a}=R_{potf}\times T_{t}\times T_{f}\times T_{df}$$ (10)

$$\text{Direct radiation}\to R_{b}=R_{potsl}\times T_{t}\times T_{f}\times \left(\text{1}-T_{df}\right)$$ (11)

Where *R*_*potf*_ is the astronomical radiation on a horizontal surface; *R*_*potsl*_ is the astronomical radiation on a slope surface; *T*_*f*_ is the transmittance effect of cloud cover; *T*_*t*_ is the clear-sky transmittance; *T*_*df*_ is the diffuse radiation fraction.

The precipitation and temperature data were derived from observations at multiple weather stations within the study area. Thiessen polygons were generated using ArcGIS based on the spatial distribution of the weather stations, and the weight of each polygon was calculated based on its area. Finally, area-weighted averages of temperature and precipitation were obtained for the study area.

vegetation growth models of woodland and grassland areas in the JRB, and the physiological and ecological parameters were categorized into two types: one for deciduous vegetation and one for evergreen vegetation. The proportions of different vegetation types in the JRB were obtained from statistical analysis of land-use maps. These proportions were then applied to weight both the observed LAI data and the simulated LAI results under the two sets of physiological and ecological parameters, resulting in the observed and simulated LAI for woodland and grassland in the basin. In addition, the model parameters were calibrated and verified using both observed and simulated LAI data for the JRB from 1998 to 2016. The determination coefficient *R*^2^ and Nash–Sutcliffe coefficient (E_NS_) were used as evaluation indices for model calibration and validation [45].


$$R^{\text{2}}={\left[\frac{\sum_{i=\text{1}}^{n}\left(Q_{m}-\overset{―}{Q_{avg}}\right){\left(Q_{p}-\overset{―}{Q_{p}}\right)}^{\text{2}}}{\sqrt{\sum_{i=\text{1}}^{n}{\left(Q_{m}-\overset{―}{Q_{avg}}\right)}^{\text{2}}}\times \sqrt{\sum_{i=\text{1}}^{n}{\left(Q_{p}-\overset{―}{Q_{p}}\right)}^{\text{2}}}}\right]}^{\text{2}}$$ (12)

$${\text{E}}_{\text{NS}}=\text{1}-\frac{\sum_{i=\text{1}}^{n}{\left(Q_{m}-Q_{p}\right)}^{\text{2}}}{\sum_{i=\text{1}}^{n}{\left(Q_{m}-\overset{―}{Q_{avg}}\right)}^{\text{2}}}$$ (13)

where $Q_{m}$ is the observed value; $\overset{―}{Q_{avg}}$ is the observed mean; $Q_{p}$is the simulated value; and $\overset{―}{Q_{p}}$ is the simulated mean.

Greater *R*^2^ values indicate a stronger correlation between the simulated series and the observed series, while greater E_NS_ values indicate higher reliability of the mean behavior of the system [46]. Typically, an *R²* value of is at least 0.6 and the value of E_NS_ is at least 0.5 to ensure simulation accuracy.

PEST (Parameter Estimation Software) is an external tool for uncertainty analysis and parameter optimization independent from Biome-BGC. It employs a combination of the nonlinear Gauss–Marquardt–Levenberg algorithm combined with gradient descent and Gauss–Newton methods. PEST offers rapid convergence and global search capabilities. It adjusts vegetation physiological parameters to minimize the difference between the model output and reference value, without modifying the model code [47–48]. In this study, the Biome-BGC model was simulated by a combination of Spin-up and Normal modes. The data of 1975 were used to initialize the Spin-up process. After 7,200 years of repeated simulation, the difference between the simulation results from two consecutive years approached zero, indicating that the model had reached a stable equilibrium state. Based on the equilibrium output of the Spin-up phase, the Normal simulation was conducted using data from 1976 to 2016. Using observed LAI data from the JRB as a reference, a combination of PEST parameter optimization and manual parameter adjustment was used to perform sensitivity analysis and calibration verification of the model. First, preliminary parameter ranges were determined based on relevant literature, and physiological and ecological parameters with high sensitivity were selected. To ensure model stability during the calibration process, all parameters were divided into groups of approximately ten, and each group was calibrated step by step. After the parameters within each group reached local optima, their values were fixed while proceeding to the next group until all groups achieved local optimization. Finally, based on the automatic calibration results, manual adjustments were conducted to further refine the parameters, and the final values of each parameter were determined individually.

### 2.4. Future climate prediction

Various Shared Socioeconomic Pathways (SSPs) in CMIP6 are an important means to predict climate change trends under different scenarios in the future. Based on relevant literature, we selected four climate models—CMC-ESM2, INM-CM4–8, MIROC6, and NorESM2-LM—which have been shown to effectively simulate climate change trends in East Asia. For each of these models, climate data under four Shared Socioeconomic Pathways—sustainable development (SSP126), moderate development (SSP245), regionally unbalanced development (SSP370), and conventional development (SSP585)—were used in this study [49]. Bias correction was conducted using power transformation for precipitation and quantile mapping was applied to for temperature downscaling. The final climate input was obtained by averaging the corrected outputs of the four GCMs [50]. This enabled the assessment of climate change in the study area during three time periods: 2023–2040 (near term), 2041–2070 (medium term), and 2071–2100 (long term). The projected precipitation and temperature data under each future scenarios were input into the calibrated Biome-BGC model to simulate vegetation growth in the basin under future climate conditions.

## 3. Results and discussion

### 3.1. Calibration and verification of vegetation growth model

**Fig 2:**
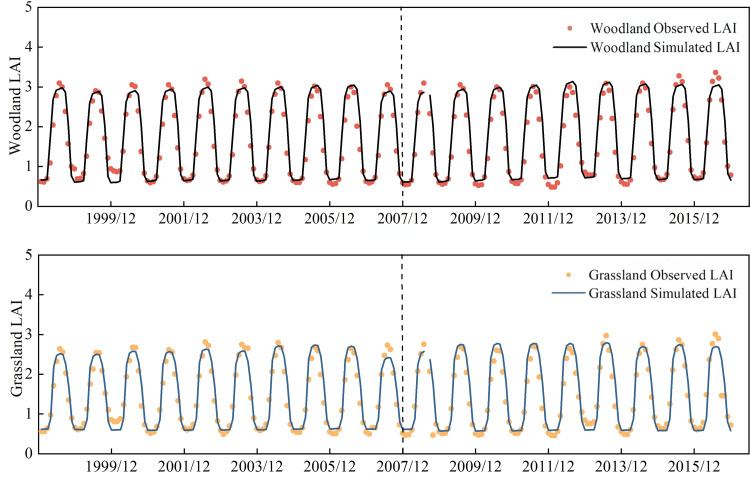
Calibration and verification results of LAI.

Sensitivity analysis of the vegetation growth model for the JRB indicated that several parameters exhibited high sensitivity, including the leaf C:N ratio, carbon allocation ratio, specific leaf area, canopy extinction coefficient, canopy interception coefficient, leaf nitrogen content in Rubisco enzyme, maximum stomatal conductance, and photosynthetic product turnover rate. The optimized values for some physiological and ecological parameters are shown in Table 3. Simulated results of the calibrated model were calculated (Table 4, Fig 2), and the LAI during the calibration period (1998–2007) and validation period (2008–2016) achieved *R*^2^ values greater than 0.8 and E_NS_ values greater than 0.7. These results indicate that the developed vegetation growth model is well-suited for simulating the vegetation dynamics of the JRB and can be reliably applied in this study.

**Table 3:** Physiological and ecological parameters of the model.

Parameter symbol	Parameter meaning	Units	Range of values
FTG	transfer growth period as fraction of growing season	prop.	0.1–0.3
FL	litterfall as fraction of growing season	prop.	0.2–0.32
LFRT	annual leaf and fine root turnover fraction	yr ^− 1^	0.1–1.0
LWT	annual live wood turnover fraction	yr ^− 1^	0–0.9
WPM	annual whole-plant mortality fraction	yr ^− 1^	0–0.005
FM	annual fire mortality fraction	yr ^− 1^	0–0.005
FRC:LC	new fine root C:new leaf C	Ratio	0.5–2.0
SC:LC	new stem C:new leaf C	Ratio	0–1.1
LWC:TWC	new live wood C:new total wood C	Ratio	0.1–0.3
CRC:SC	new root C:new stem C	Ratio	0–0.23
CGP	current growth proportion	prop.	0.15–0.5
C:N_leaf_	C:N of leaves	kgC kgN ^− 1^	15–30
C:N_lit_	C:N of leaf litter	kgC kgN ^− 1^	40–97
C:N_fr_	C:N of fine roots	kgC kgN ^− 1^	42–57
C:N_lw_	C:N of live wood	kgC kgN ^− 1^	0–97
C:N_dw_	C:N of dead wood	kgC kgN ^− 1^	0–398
L_lab_	leaf litter labile proportion	DIM	0.32–0.39
L_cel_	leaf litter cellulose proportion	DIM	0.44
L_lig_	leaf litter lignin proportion	DIM	0.17–0.24
FR_lab_	fine root labile proportion	DIM	0.22–0.3
FR_cel_	fine root cellulose proportion	DIM	0.3–0.45
FR_lig_	fine root lignin proportion	DIM	0.25–0.48
DW_cel_	dead wood cellulose proportion	DIM	0.66–0.75
DW_lig_	dead wood lignin proportion	DIM	0.25–0.34
W_int_	canopy water interception coefficient	LAI ^− 1^·d ^− 1^	0.021–0.045
K	canopy light extinction coefficient	DIM	0.51–0.65
LAI_all:proj_	all-sided to projected leaf area ratio	DIM	2.0–2.6
SLA	canopy average specific leaf area ratio	m^2^·kgC ^− 1^	20–60
SLA_shade:sun_	ratio of shaded SLA: sunlit SLA	DIM	1.9–2.0
FLMR	fraction of leaf N in Rubisco	DIM	0.07–0.1
g_smax_	maximum stomatal conductance	m·s ^− 1^	0.005–0.01

**Table 4:** Model calibration and verification results.

Evaluation index	Woodland		Grassland	
	calibration	verification	calibration	verification
*R* ^2^	0.86	0.89	0.92	0.89
E_NS_	0.84	0.77	0.90	0.86

### 3.2. Future changes in basin climate

Based on the observed precipitation and temperature data from meteorological stations in the JRB from 1975 to 2016, power transformation for precipitation and quantile mapping for temperature downscaling were used to calibrate four GCMs, namely CMCC-ESM2, INM-CM4–8, MIROC6, and NorESM2-LM. The evaluation results are presented in Table 5; the mean error of simulated precipitation after downscaling of the four selected GCMs was less than 6%, and the mean error of simulated temperature was less than 0.67 °C, which meets the accuracy requirements of the study.

**Table 5:** Average errors of precipitation and temperature for each climate model compared with historical data (1975–2016) (precipitation: annual total; temperature: multi-year mean).

GCM	Precipitation(%)	Maximum temperature(°C)	Minimum temperature(°C)
CMCC-ESM2	−3.6 ± 2.00	0.007 ± 0.77	0.008 ± 0.47
INM-CM4–8	0.53 ± 2.15	0.014 ± 0.74	0.014 ± 0.52
MIROC6	5.90 ± 2.07	−0.003 ± 0.93	0.001 ± 0.53
NorESM2-LM	3.26 ± 1.39	−0.011 ± 0.83	−0.004 ± 0.54

**Fig 3:**
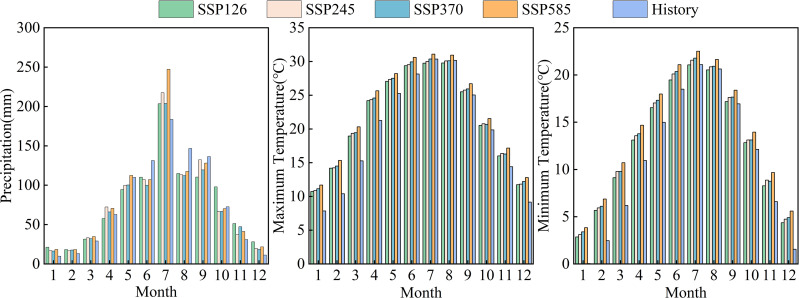
Comparison of monthly mean precipitation and temperature between historical data (1975–2016) and different Shared Socioeconomic Pathways (SSPs, 2023–2100).

To reduce the uncertainty caused by differences among individual GCMs, we employed a multi-model ensemble averaging approach. The average of the simulation outputs from the four GCMs under different scenarios was used as the projected future climate for the study area. Compared with that during the historical baseline period (1976–2016), the JRB is projected to experience a wetter and warmer climate during the upcoming three periods (Table 6, Fig 3). In terms of precipitation changes, the near-term precipitation under the SSP245 and SSP370 scenarios was slightly lower than that during the baseline period, but over time, the rainfall in the basin under all four scenarios showed a gradual increase, with the largest increase under the SSP585 scenario, in which rainfall was projected to increase by 27.33% compared with that in the baseline period by the end of the 21st century. In terms of temperature change, the minimum and maximum temperatures of the basin in different periods under the four scenarios were projected to rise to different degrees, with the temperature rise of the SSP585 scenario being the largest, having projected increases by the end of the 21st century of 3.9 °C and 4.0 °C for maximum and minimum temperatures, respectively, compared with those for the baseline period. In terms of annual climate change, the distribution of precipitation and temperature in all scenarios exhibited substantial seasonal differences while maintaining synchronized variation trends in precipitation and temperature. As shown in Fig 3, precipitation was mainly concentrated from May to September, with peak rainfall in July and the lowest levels in February. Although temperature change was relatively uniform, there was a steady increase from January to July, peaking mid-year and then gradually declining. The precipitation concentrated in summer is expected to enhance seasonal contrasts in the future climate. On the one hand, these seasonal differences may promote the vigorous growth of vegetation in summer, and on the other hand may also have an impact on the runoff production, confluence and erosion of the underlying surface.

**Table 6:** Changes in precipitation and temperature from 2023 to 2100 compared with the findings for the baseline scenario.

Climate scenario	Period	Variation of precipitation (%)	Variation of maximum temperature (°C)	Variation of minimum temperature (°C)
SSP126	2023–2040	3.07%	0.9	0.8
	2041–2070	8.00%	1.5	1.4
	2071–2100	10.50%	1.7	1.5
SSP245	2023–2040	−2.48%	0.8	0.9
	2041–2070	8.67%	1.6	1.7
	2071–2100	11.08%	2.2	2.3
SSP370	2023–2040	−6.71%	0.5	0.7
	2041–2070	2.47%	1.6	1.6
	2071–2100	6.53%	2.8	2.9
SSP585	2023–2040	0.87%	1.1	1.0
	2041–2070	6.88%	2.3	2.3
	2071–2100	27.33%	3.9	4.0

Different climate emission pathways have distinct impacts on the hydrological balance and disaster mitigation priorities within the study area. Under the low-emission pathway (SSP126), temperature increases are limited, and precipitation changes are moderate, resulting in a relatively stable hydrological cycle. In this scenario, the risk of ecological disasters is low, and maintaining good vegetation cover can help prevent soil erosion and flooding. Under the medium-emission pathways (SSP245 and SSP370), warming and precipitation trends tend toward extremes, and increased evapotranspiration leads to heightened seasonal water stress. This places higher demands on water balance management, necessitating measures such as water-saving agriculture and ecological regulation to mitigate the risks of floods and landslides. Under the high-emission scenario (SSP585), both the frequency and intensity of extreme climate events increase significantly, shifting the regional water balance toward deficit. This elevates the overall risk of floods and debris flows, posing substantial challenges to watershed disaster mitigation and water resource management.

### 3.3. Vegetation dynamics in the basin under different climatic scenarios

#### 3.3.1. Vegetation dynamics from 1976 to 2016.

**Fig 4:**
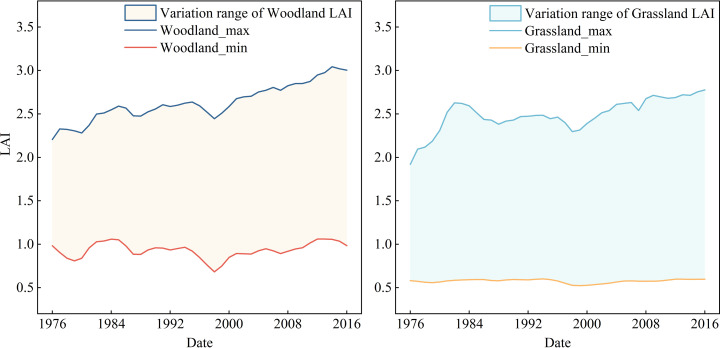
Interannual variations in maximum and minimum LAI of woodland and grassland during 1976–2016.

Simulation results of the vegetation growth in the JRB indicate that the annual trend in LAI for both woodland and grassland was relatively consistent from 1976 to 2016. These trends mirrored the variation in precipitation over the same period, showing a fluctuating upward trend, with more pronounced increases in woodland. In 1997–1998, the LAI values of woodland and grassland declined relative to adjacent years. This was primarily due to a significant reduction in precipitation over the basin during this period (with an annual total precipitation of 651.1 mm in 1997, the lowest between 1975 and 2000) and an increase in temperature (with an annual mean maximum temperature of 20.8 °C in 1998, the highest within the same period). These conditions reduced water allocation and uptake by vegetation leaves and roots, ultimately suppressing growth and leading to a decline in LAI. This also showed that vegetation changes are strongly influenced by climatic factors. The annual maximum LAI ranged approximately from 2.21 to 3.04, while the minimum LAI ranged from 0.68 to 1.06. For grassland, the annual maximum LAI ranged from 1.92 to 2.78, and the minimum ranged from 0.52 to 0.60 (Fig 4). The overall LAI level of woodland was higher than that of grassland, indicating that the area of leaves that could participate in photosynthesis in woodland was larger, with lusher vegetation growth.

**Fig 5:**
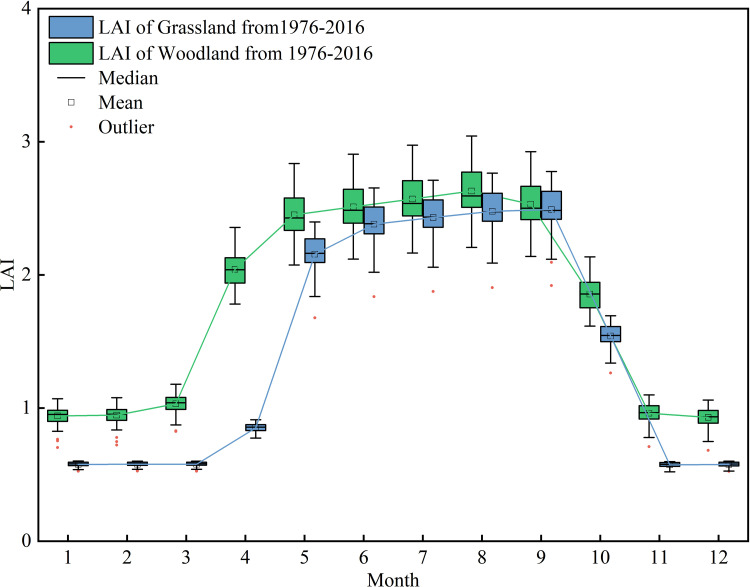
Simulation results of changes in monthly mean LAI, averaged over the period 1976 to 2016.

The simulation results of the monthly average LAI from 1976 to 2016 indicated that both woodland and grassland exhibited significant annual variations in LAI, with similar seasonal trends (Fig 5). From January to March, the temperature in the basin was low and precipitation was limited. Woodland LAI remained below 1.2, and grassland LAI was below 0.7, both remaining at relatively low levels, indicating impoverishment of regional vegetation. With rising temperature and increasing precipitation from April onward, vegetation entered a vigorous growth phase. Woodland LAI peaked 3.04 in August, while grassland LAI reached 2.78 in September. Moreover, higher box height from May to September indicated that the differences in vegetation growth of different years were mainly reflected in summer, whereas vegetation cover remained relatively consistent during the dormant period of each year. This further demonstrated that the weather was the primary drivers of vegetation growth. After reaching their peak levels, as temperature and precipitation both declined, LAI for both vegetation types decreased sharply, which would result from the photosynthetic products in the vegetation being sequestered in roots and stems, while the leaves were gradually shed and decomposed by soil microorganisms. The coefficient of variation of mean monthly LAI was 0.42 for woodland and 0.60 for grassland, suggesting that woodland LAI fluctuated less throughout the year, while more stable plant growth, whereas grassland LAI exhibited greater intra-annual variation and was more sensitive to external environmental factors. This is because grasslands are primarily composed of annual herbaceous plants with short growth cycles and strong seasonality. They grow rapidly in spring and summer but wither quickly in autumn and winter, making them highly sensitive to short-term fluctuations in precipitation and temperature, which leads to pronounced seasonal variability in LAI [51]. In contrast, woodlands are dominated by trees with deep root systems and long lifespans, allowing them to access deeper soil moisture and buffer against short-term weather anomalies more effectively [52].

#### 3.3.2. Impacts of future climate change on vegetation in the basin.

**Fig 6:**
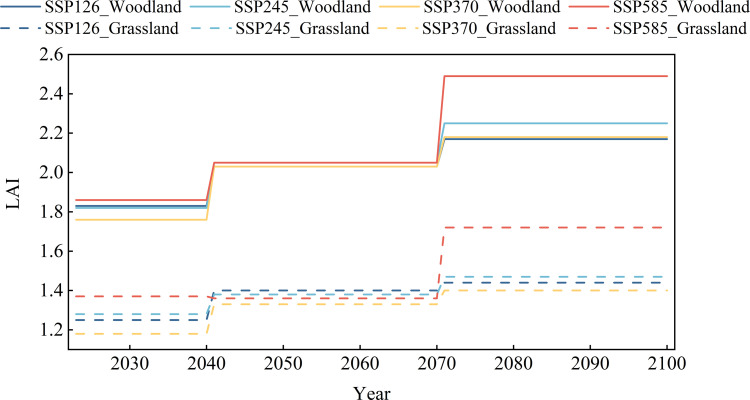
Predicted interannual changes of mean LAI for woodland and grassland under different Shared Socioeconomic Pathways from 2023 to 2100.

**Fig 7:**
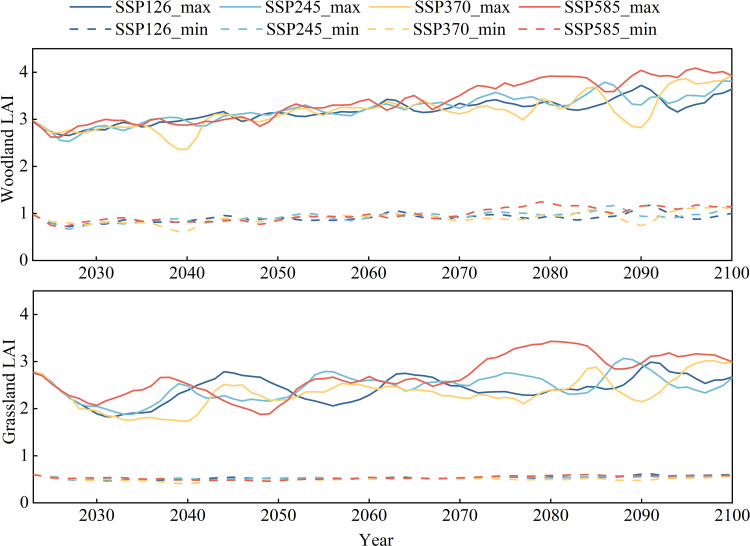
Predicted interannual variations of maximum and minimum LAI of grassland and woodland under different Shared Socioeconomic Pathways during 2023–2100.

Based on four climate change scenarios and the established vegetation growth model, the meteorological driving data of the Biome-BGC and MT-CLIM models were updated to simulate vegetation cover changes in the JRB under different scenarios (Fig 6, Fig 7). Under all four scenarios, LAI of both woodland and grassland exhibited increasing trends from 2023 to 2100. Vegetation cover was projected to be highest under the SSP585 scenario, with an average LAI of 2.49 for woodland, an increase of 22.5% compared with the average LAI from 1976–2016, with a peak value of 4.09. For grassland, the average LAI was 1.72, representing a 4.9% increase compared with the 1976–2016 baseline, with a maximum LAI of 3.43. From 2023 to 2100, the increases in woodland LAI under different climate scenarios are projected to be 18.6% (SSP126), 23.6% (SSP245), 23.9% (SSP370), and 33.9% (SSP585), respectively; for grassland, the corresponding increases are 15.2% (SSP126), 14.8% (SSP245), 18.6% (SSP370), and 25.5% (SSP585). Under the low-emission scenario (SSP126), changes in regional vegetation cover are relatively moderate, whereas under the high-emission scenario (SSP585), both woodland and grassland LAI show significant increases, with woodland exhibiting a greater magnitude of growth than grassland. This indicates that a warmer and more humid future climate, along with elevated atmospheric CO2 concentrations, will be more favorable for the expansion of leaf area in both woodland and grassland, which is consistent with the predicted climate change trends. However, the LAI of grassland under all four scenarios was slightly lower than that during the baseline period in the near term. This outcome may be due to the substantial increase in temperature that alters the evapotranspiration and water cycling processes in the basin, as well as insufficient precipitation recharge, which affects canopy interception, root absorption, transpiration of vegetation, and decomposition of litter in the soil, thereby affecting the growth of vegetation and resulting in a slight decrease in vegetation cover [53,54]. In terms of annual patterns, LAI showed substantial seasonal differences in both woodland and grassland in the future. The vegetation in both woodland and grassland entered the vigorous growth period in April and maintained relatively high coverage throughout the summer, beginning to decline gradually in October. Compared with that of woodland, the vegetation of grassland with poor stability had more significant differences between growth and dormancy periods, consistent with baseline patterns.

In addition, regarding the impact of extreme climate events, the Biome-BGC model can indirectly capture the cumulative physiological effects of such events through processes regulated by water stress, temperature limitations, and solar radiation. For example, in our simulation, the decline in LAI during 1997–1998 reflected a regional drought, even though no explicit event tagging was applied. However, it is important to acknowledge that certain event-specific impacts—such as plant mortality following flooding or physiological damage induced by heatwaves—are not represented in the Biome-BGC model. Therefore, when ecosystem dynamics are primarily driven by extreme events, the model may underestimate their impacts. This limitation will be addressed in future research efforts.

The results of this study indicate that under projected climate warming, vegetation cover in the study area is expected to increase substantially, with significant implications for regional water balance, carbon cycling, and land-use management. Enhanced vegetation cover is likely to increase regional evapotranspiration, potentially leading to seasonal water shortages and posing challenges for water management strategies [55,56]. Furthermore, the continued increase in LAI is generally associated with higher photosynthetic rates and greater biomass accumulation, thereby enhancing ecosystem carbon storage and contributing to the maintenance of regional carbon balance [57]. Changes in LAI also have important implications for regional land-use planning, as future management should account for the allocation of cropland, grassland, and woodland to optimize key ecosystem services, including soil conservation and climate regulation. Overall, this study reveals the projected trends of vegetation cover in the JRB, providing a basis for predicting ecosystem responses to climate change and offering scientific reference for future water management.

## 4. Conclusions

Using the Jialing River Basin as a case study, this research predicted the future climate change in the basin under different scenarios, constructed growth models for different types of vegetation in the JRB, analyzed the change in vegetation cover in the basin and its projected response to climate change. The main conclusions are as follows:

(1) The *R*^2^ and E_NS_ values of LAI simulated by our Biome-BGC model during the calibration and validation periods met all the requirements for model adequacy. The optimal ranges of various physiological and ecological parameters of different vegetation in the basin were determined by identifying those parameters with high sensitivity through PEST and further fine-tuning them manually. The vegetation growth model thus established has good applicability for different vegetation types in the JRB and can be used for simulating vegetation growth under different scales of basin environmental change.
(2) The model predicted that the climate of the Jialing River Basin will maintain a warm and humid condition with concurrent rain and heat in the future. Both temperature and precipitation in the JRB displayed fluctuating upward trends under the four climate change scenarios (SSP126, SSP245, SSP370 and SSP585) until the end of this century. The change under the SSP585 scenario was the most substantial, with the greatest maximum increases for both temperature and precipitation.
(3) There were significant differences in LAI of vegetation in the Jialing River Basin under different climatic conditions. The vegetation cover of both woodland and grassland in the basin remained at a high level from 1976 to 2016. Peak growth periods of vegetation were closely related to summertime precipitation and temperature conditions, and on a monthly timescale, grassland vegetation was more sensitive to climate variability. Our model projects that although the LAI of the basin’s vegetation is expected to initially decrease slightly compared with the historical period, increasingly warm and humid climate will promote vegetation growth, and the LAI of different vegetation types is expected to show a fluctuating growth trend from 2023 to 2100 under all four predicted climate change scenarios. The change in vegetation was the most sizable under the SSP585 scenario, with the largest increases in LAI for both woodland and grassland environments. In the context of long-term changes, woodland exhibits greater potential for LAI accumulation than grassland.
(4) The future directions of this study are as follows: 1) coupling the model with more sophisticated climate event-driven modules or integrating it with disaster impact models to enhance its capacity for simulating vegetation responses under extreme climate events and 2) utilizing long-term land use datasets to analyze vegetation evolution under the combined influences of climate variability and underlying surface changes.

## Supporting information

S1 File Climate Data. (XLSX)

S2 File LAI Simulation Data. (XLSX)

S3 File Future LAI Data. (XLSX)

S4 File Supplementary information. (DOCX)
