# Supplementary material for: Impacts of climate change on basin vegetation based on Biome-BGC model: A case study with the Jialing River Basin
Source: PLoS One. 2026 Feb 13;21(2):e0335685. doi: 10.1371/journal.pone.0335685 (PMC12904437; doi:10.1371/journal.pone.0335685)
Supplement: S4 File — (DOCX) [file pone.0335685.s004.docx]

**Supplementary Information**

**Table S1. Prediction of mean LAI under future climate**

| Climate scenario | Period | Woodland | Grassland |
| --- | --- | --- | --- |
| SSP126 | 2023–2040 | 1.83 | 1.25 |
|  | 2041–2070 | 2.03 | 1.40 |
|  | 2071–2100 | 2.17 | 1.44 |
| SSP245 | 2023–2040 | 1.82 | 1.28 |
|  | 2041–2070 | 2.05 | 1.38 |
|  | 2071–2100 | 2.25 | 1.47 |
| SSP370 | 2023–2040 | 1.76 | 1.18 |
|  | 2041–2070 | 2.03 | 1.33 |
|  | 2071–2100 | 2.18 | 1.40 |
| SSP585 | 2023–2040 | 1.86 | 1.37 |
|  | 2041–2070 | 2.05 | 1.36 |
|  | 2071–2100 | 2.49 | 1.72 |
